# Rapid review programs to support health care and policy decision making: a descriptive analysis of processes and methods

**DOI:** 10.1186/s13643-015-0022-6

**Published:** 2015-03-14

**Authors:** Julie Polisena, Chantelle Garritty, Chris Kamel, Adrienne Stevens, Ahmed M Abou-Setta

**Affiliations:** Canadian Agency for Drugs and Technologies in Health, 600-865 Carling Avenue, Ottawa, Ontario K1S 5S8 Canada; Department of Epidemiology and Community Medicine, Faculty of Medicine, University of Ottawa, 451 Smyth Road, Ottawa, Ontario K1H 8 M5 Canada; Knowledge Synthesis Group, Ottawa Hospital Research Institute, 501 Smyth Road, Ottawa, Ontario K1H 8 L6 Canada; George and Fay Yee Centre for Healthcare Innovation, University of Manitoba/Winnipeg Regional Health Authority, GE-706 – 820 Sherbrook Street, Winnipeg, Manitoba R3A 1R9 Canada

**Keywords:** Rapid views, Systematic reviews, Evidence synthesis, Health care decision making

## Abstract

**Background:**

Health care decision makers often need to make decisions in limited timeframes and cannot await the completion of a full evidence review. Rapid reviews (RRs), utilizing streamlined systematic review methods, are increasingly being used to synthesize the evidence with a shorter turnaround time. Our primary objective was to describe the processes and methods used internationally to produce RRs. In addition, we sought to understand the underlying themes associated with these programs.

**Methods:**

We contacted representatives of international RR programs from a broad realm in health care to gather information about the methods and processes used to produce RRs. The responses were summarized narratively to understand the characteristics associated with their processes and methods. The summaries were compared and contrasted to highlight potential themes and trends related to the different RR programs.

**Results:**

Twenty-nine international RR programs were included in our sample with a broad organizational representation from academia, government, research institutions, and non-for-profit organizations. Responses revealed that the main objectives for RRs were to inform decision making with regards to funding health care technologies, services and policy, and program development. Central themes that influenced the methods used by RR programs, and report type and dissemination were the imposed turnaround time to complete a report, resources available, the complexity and sensitivity of the research topics, and permission from the requestor.

**Conclusions:**

Our study confirmed that there is no standard approach to conduct RRs. Differences in processes and methods across programs may be the result of the novelty of RR methods versus other types of evidence syntheses, customization of RRs for various decision makers, and definition of ‘rapid’ by organizations, since it impacts both the timelines and the evidence synthesis methods. Future research should investigate the impact of current RR methods and reporting to support informed health care decision making, the effects of potential biases that may be introduced with streamlined methods, and the effectiveness of RR reporting guidelines on transparency.

**Electronic supplementary material:**

The online version of this article (doi:10.1186/s13643-015-0022-6) contains supplementary material, which is available to authorized users.

## Background

Systematic reviews (SRs) are accepted as the gold standard in evidence-based medicine. Health care decision makers, however, are increasingly in need of evidence-based reports in limited timeframes to support informed decisions [1]. This has led to the evolution of rapid reviews (RR), with no common description of their purpose, methods, and format as they vary in time to completion, report format, literature search strategies, and methods used for evidence synthesis [1,2]. RR can be described as evidence syntheses that use methods to streamline those of SRs to complete the evidence synthesis in a shorter turnaround time [1,3]. Watt et al. concluded that while RRs are instrumental in answering specific policy questions, they should not be used to replace traditional SRs. Although their comparisons were a small sample of reports, it was suggested that rapid reviews should be ‘fit for purpose’ and tailored to the knowledge needs of the commissioning body and/or the intended end users. Further, rapid reviews should not be viewed as inherently inferior to full systematic reviews but that more evidence is needed to inform their methodology and application [2]. Furthermore, Watt et al. [2] found that the overall conclusions did not differ much between RRs and traditional SRs for similar research questions. On the other hand, SRs were more likely to incorporate in-depth results on clinical outcomes, economic considerations, and health service impact. While a standardized RR methodology may not be considered feasible, a need for methodological research and increased transparency in reporting has been identified [4].

Decision makers are increasingly seeking evidence to inform the policymaking process, and this requires access to high-quality evidence to inform decisions. As such, several health care organizations have turned to abbreviated systematic methods as a means of delivering evidence in a timely manner. One of the major issues with RRs, however, is that their methodologies are often not published or available outside the organization conducting the RR. To date, we have identified two publications that describe RR programs in detail [3,5], while it is estimated that conducting RRs is widespread and an integral part in health care policy and decision making. Moreover, RRs are not without their challenges. First and foremost, it is not known how many health care organizations undertake rapid reviews or what methods they use. Even less well understood is when to undertake RRs, whether RRs provide similar results to SRs, and what potential bias is introduced when abbreviating the SR review approach.

In September 2013, a group of three health care research organizations with a shared interest in RRs organized and facilitated an open ‘Rapid Review Methods Discussion’ at the 2013 Cochrane Colloquium in Quebec City. Among the 40 meeting registrants, academia, government, research institutions, not-for-profit organizations, and the Cochrane Collaboration were represented. The primary purpose was to exchange and share information between RR producers and users about the methods used to develop RRs and their usability and appropriateness to support informed decisions in health care. One of the key outcomes of this meeting was establishing an informal network of organizations and researchers interested in ‘rapid reviews’. Further, recognizing the growing need to better understand the methods used by rapid review producers, a number of organizations agreed to share their processes and methods. The purpose of this study, hence, was to describe the various RR programs with the aim to provide an analysis of processes and methods by highlighting themes of characteristics across with these rapid review programs. Our descriptive analysis represents the most comprehensive attempt to date to characterize a broad spectrum of RR programs and their respective methods. The discussion reflected a wide ranging set of views on what methods should be maintained and what can be sacrificed for the sake of timeliness.

## Methods

### Study sample

Following the rapid review methods discussion meeting at the 2013 Cochrane Colloquium, we applied purposive and snowball sampling techniques to form our study sample. Meeting participants were approached to share information about the methods and processes used in their RR programs. Additional RR programs were identified by the participants and this study’s coauthors. The Canadian Agency for Drugs and Technologies in Health (CADTH) led the environmental scan of international rapid review programs with contributions from the Ottawa Hospital Research Institute (OHRI) and University of Manitoba. As CADTH is an independent, not-for-profit organization funded by Canadian federal and provincial governments, we are not required to seek ethics approval to obtain information from various organizations about their practices and methods on a specific health care topic. In our correspondence with the organization, we indicated a priori that any information provided will be used in a publication, so consent to publish was not needed as the feedback was provided on a voluntary basis.

### Data collection

A form was developed and organized into nine domains to collect information specific to methods and processes by the identified program (Table S1 in Additional file 1). The participants were asked to provide details about their program’s definition of RR, report types produced by their program, details regarding topic selection, education sessions with client or funder, protocol development, report production, submission and dissemination, if the report becomes publicly available and if there was a website for their RR program. Details for the following RR programs were pre-populated to illustrate the types of responses requested from the participants: the CADTH, Oregon Health Sciences University, and OHRI.

### Data analysis

Results were collected on a voluntary basis and collated in an electronic database for review, quality assurance, and analysis. The unit of analysis was the RR program and not the type of RR produced as some programs provide a range of RR services. We calculated the frequencies of responses for questions with predefined answers. For the remaining questions, we listed program responses as there was potential for misinterpretation given the array of answers. The characteristics associated with the processes and methods for the RR programs in our sample were summarized narratively according to their responses. Subsequently, we compared and interpreted the results to identify the underlying themes and patterns from the RR program descriptions.

## Results

### Countries of origin

Twenty-nine RR programs were included in our sample. Twelve RR programs were based in Canada, five from the USA, three each from Australia and UK, two each from Germany and Finland, and one each from Italy and Taiwan. Our sample included broad organizational representation from academia, government, research institutions, and non-for-profits.

### Rapid review definition

Our responses revealed that there was no standard definition for RR. Definitions were organized into six categories based on the descriptions provided. They are as follows: accelerated, condensed, focused, type of evidence synthesis, modified systematic review, and tailored. Individual responses are presented in Table S2 in Additional file 2.

### Purpose of rapid review report

Responses from the participants indicated that the primary objectives to produce RRs were to inform decision making with regards to funding health care technologies, services, and policy and program development. Some decision makers requested evidence for clinical decision making, implementation, clinical and operational effectiveness, and efficiency. In addition, these reviews were reported to be used to complement discussions between health service staff and manufacturers and to improve the understanding of a specific topic based on the published evidence.

### Types of report produced by rapid review programs

There was a wide array of RR report types in our sample (Table S3 in Additional file 3). Report types can range from a simple annotated bibliography to a rigorous health technology assessment (HTA) of a single health technology. The full spectrum included reference lists, summaries of abstracts, reviews of reviews, rapid systematic reviews, systematic reviews and meta-analyses, and single HTA. Several organizations produce more than one type of RR.

### Processes

#### Development of protocol

Most RR programs (*n* = 28; 96.6%) incorporated protocol development as part of the RR process; one respondent did not indicate if a protocol is used or not. Components specified in a protocol centered mainly on patient population(s), intervention(s), comparator(s), outcome(s) and study design(s), and the research question(s). Fifteen RR programs (51.7%) prepared protocols in conjunction with the requestor.

#### Review process of draft report

Twenty-four RR programs (82.8%) reported conducting internal reviews of the draft RR and a subset of programs (*n* = 13; 44.8%) also had drafted RRs reviewed externally. Seven respondents (24.1%) stated that a decision to carry an external review depended on the type of RR report and timeline for completion, and additionally only if requested. In two instances, it was reported that the requestor regularly reviewed the draft report before being finalized. Information from industry was generally not sought, with only a handful of respondents indicating that they do this on a regular basis (*n* = 4; 13.8%) or on a case-by-case basis (*n* = 3; 10.3%).

#### Report template

Based on responses from our sample, elements consistently incorporated in a RR based on the study sample are key messages or recommendations to support dissemination (*n* = 28; 96.6%), context and policy issues/implications of the synthesized evidence (*n* = 26; 89.7%), references to review methods used (*n* = 26; 89.7%), and an explanation of the strengths and limitations of review methods used (*n* = 26; 89.7%). Some RR producers also included tables (*n* = 18; 62.1%), graphs (*n* = 15; 51.7%), and a legal disclaimer (*n* = 12; 41.4%) in the final report.

#### Turnaround time for final report

The turnaround time to produce a RR ranged from 1 week to 12 months; median was 3 months. The timeframe is reflective of the differing interpretation of ‘rapid’ to various organizations. Although we did not cross reference each report type with the turnaround time for report completion, we can assume that the length of time was dependent on the complexity of the research topic, analyses required to synthesize the evidence, and types of reports produced.

#### Rapid review dissemination

Twenty RR producers (69.0%) disseminated their report beyond the requestor, while five (17.2%) responded that dissemination depends on the sensitivity of the topic and/or permission from the requestor. In addition, 11 RR producers (37.9%) posted the full rapid review on the Internet, with one additional producer reporting just posting a summary of the report. Three respondents indicated that the Web posting of their RR depended on the type of report produced and/or if permission was granted by the requestor. Table S3 in Additional file 3 lists tools commonly used by RR producers to disseminate their reports to a wider audience. Common examples include stakeholder meetings or workshops, presentations at academic or policy conferences and forums, the use of social media (e.g., Twitter and RSS) and video summaries, webinars, publication in peer-reviewed journals, online review summary databases and newsletters, email distributions, and preparation of summaries and other supporting materials.

### Methods used

#### Types of research questions

For many programs, requestors sought evidence on clinical effectiveness (*n* = 16; 55.2%), clinical efficacy (*n* = 12; 41.4%), cost-effectiveness and/or cost savings (*n* = 12; 41.4%), safety (*n* = 9; 31.0%), and to support clinical practice guideline preparation (*n* = 5; 17.2%) for either a health care technology or service. Nine respondents (31.0%) also stated that research questions focused on the accuracy of diagnostic or screening tests. Some RR programs focused exclusively on questions centered on health care policy, coverage of a technology, health system interventions, health services delivery, operational efficiency, and quality improvement. Two organizations (6.9%) focused on specific health topics. More specifically, one RR program focused exclusively on questions related to HIV/AIDS and other sexually transmitted and blood borne infections, and another focused on topics related to public health programs. One RR program in Canada was focused on health and social science topics associated with suicide prevention, conceptions of masculinity, obesity, and musculoskeletal injuries.

#### Literature search strategy

All RR programs incorporated multiple databases and websites as data sources for their reports. Commonly searched databases included PubMed/MEDLINE, CINAHL, Embase, PsycINFO, Cochrane Library, and University of York’s Centre for Reviews and Dissemination and TRIP databases. In our study, grey literature was defined as literature that is not commercially available or indexed by major databases. Fourteen RR producers (48.3%) reported consistently searching trial registries and medical society, government regulatory, and health technology assessment websites for relevant grey literature (Table S4 in Additional file 4). Twelve RR producers (41.3%) searched systematic reviews and primary studies, while 11 programs (37.9%) expanded their search strategy to incorporate primary studies if no systematic reviews were found and one (3.5%) searched primary studies if the SRs were published 2 years ago or more. Three RR programs (10.3%) did not search for primary studies, and two (6.9%) did not indicate if they searched primary studies. The search timeframe varied among producers from 5 (*n* = 5; 17.2%) to 10 years (*n* = 4; 13.8%), one producer noted searching from the year 2000 onwards. Eight participants (27.6%) responded that the time period searched was based on the topic. Literature search strategies were not peer-reviewed among 14 RR producers (48.3%), and four respondents (13.8%) indicated that it was conducted depending on the report complexity and funding available. Language restrictions were imposed by the majority of RR producers in our study, where English only studies (*n* = 17; 58.6%) or studies published in English and another language (*n* = 5; 17.2%) were searched. Seven respondents (24.1%) did not specify whether they used language restrictions as part of their search strategy.

#### Study selection and data abstraction

Numerous RR programs in our sample used two reviewers to select studies for inclusion (*n* = 16; 55.2%) and for data abstraction (*n* = 14; 48.3%). A few respondents stated that the number of reviewers for the study selection (*n* = 2; 6.90%) and data abstraction (*n* = 3; 10.3%) was predicated on the research topic.

#### Evidence synthesis

A narrative summary was the most common method used to synthesize the evidence (*n* = 27; 93.1%), while four (13.8%) reported regularly conducting a meta-analysis and three producers (10.3%) include economic evaluations in their reports. The appropriateness of a meta-analysis in a RR would partially depend on the type of report produced. Two RR programs (6.9%) did not specify the type of evidence synthesis conducted. A few RR programs occasionally performed meta-analyses (*n* = 5; 17.2%) or syntheses of the economic literature (*n* = 4; 13.9%).

#### Critical appraisal

Most RR producers (*n* = 20; 69.0%) indicated that they critically appraised the selected studies in their reports, while two (6.90%) did it depending on the report type and time permitted. Seven RR programs (24.1%) do not conduct a critical appraisal of studies. Similar to other forms of evidence synthesis or HTA, validated critical appraisal tools were selected according to their appropriateness for the study design (Table S4 in Additional file 4).

### Interpretation

Based on a sample of 29 RR programs, the study findings revealed that the definition of, and methods to conduct, RRs was more fluid and flexible compared with those established for a traditional systematic review or HTA. This phenomenon can be related in part to the novelty of RR methods versus other evidence-based approaches, tailoring of RRs to meet the decision makers’ needs, and how an organization defines ‘rapid’ since the definition impacts both the timelines and the conduct of the evidence synthesis. Central themes to factors that appear to influence the methods used by RR programs and their respective report types and dissemination are the imposed turnaround time to complete a report; resources available; the complexity and sensitivity of the research topics; and permission from the requestor. From the responses we received, it is evident that a ‘one-size fits all’ approach may not be appropriate as RRs are produced for decision makers in an array of health care areas for different purposes and with diffing available resources and time constraints. It, therefore, seems challenging to develop a formulaic process or methodology that would be endorsed by all RR producers and users. Even so, further research may reveal particular common points of interest that would allow some standardization of the methods used to conduct specific types of RR and how to best report their results.

## Discussion

Although our sample was not exhaustive, we sought to identify and synthesize the processes and methods used by RR programs throughout the world. Overall, our results were consistent with the findings of previous reviews on methods used to produce RRs [2,6]. A review of 49 RRs located in the HTA database of the Cochrane Library and six international HTA databases from 2000 onwards found many differences in literature search strategies, quality assessment, data extraction, synthesis methods, report structure, and number of reviewers conducting the RR [6]. The authors also reported a positive correlation between the length of time to produce a RR and the application of standard SR reporting methods. In another study, Watt et al. [2] surveyed 23 HTA producers (response rate = 46%) on their practices in preparing RRs. Similar to our study results, the authors did not observe any consistent trends associated with the contents, methods, and definition of a RR. Subsequently, they concluded that while it may not be possible to standardize RR methods, HTA producers should increase the transparency of the methods used in each review.

RR producers are faced with a conundrum, having to meet the time-sensitive needs of decision makers, while at the same time attempting to maintain methodological rigor. This challenge has led different research groups to routinely short-cut different aspects of the traditional review methods in order to complete the evidence synthesis in the allotted time. It is understood that the streamlined methods used to produce RRs can inadvertently introduce bias into the review process. The comprehensiveness of a RR can be reduced if the literature search strategy incorporates an insufficient number of databases and languages, searches for publications are conducted within shortened timeframes and excludes the grey literature; resulting in an increased risk of missing publications of relevancy. Although potential biases in RRs have not been empirically studied, potential sources relate to the study selection and data extraction by one reviewer. As found in both our study and noted by Watt et al. [2], RR methods can omit external peer-review by content or methodological experts of the draft report. In instances where a quality assessment of studies is not performed, decision makers are left to determine if the study results are valid or are to be interpreted with caution. Since it is becoming more common to use RRs to support health care decision making, there has been growing interest in exploring further methods research within this realm. One avenue under consideration is the establishment of a Cochrane Rapid Reviews Methods Group. The primary function of this group would be to initially serve as a forum for discussion, and information sharing with regards to RR methods across the collaboration and beyond. The ideal aim will be to further investigate whether potential biases are inherent in RRs.

RRs may be used to inform specific health care decisions in a timely manner using relevant evidence, but their appropriateness in health care and HTA merits further investigation [2]. Legal implications associated with the use of RRs for decision making also must be considered [1-4,7]. Although previous studies reported that the majority of RRs focused on the clinical effectiveness and efficacy of a heath care technology or an intervention, 12 RR producers in our sample also incorporated evidence on cost-effectiveness and/or cost-savings. This may reflect an evolution of RR methods as well as the economic considerations incorporated more frequently in health care decisions. As reported in the previous studies, most RR programs did not mention discussions regarding the impact of their reviews on health services, such as ethical, legal, and psychosocial implications.

### Limitations

Interpretation and application of the findings of this study may be influenced by several limitations. Twelve of the 29 RR programs were based in Canada, and over 70% represented English-speaking jurisdictions. Our respondents, however, represented a cross section of RR programs in existence for health care decision making today. In addition, purposive sampling was employed, so the participants were not randomly selected. To help strive for comprehensiveness in our sample, RR programs from four continents, at various levels in the health care systems with diverse foci in across health sectors provided data. An additional limitation was that we were unable to distinguish any patterns between the purpose of the RR and the methods applied to produce a report. Although we inquired about the types of research questions addressed in a RR, we were unable to assess their scope according to the responses provided. Further, we were unable to explain potential reasons and causes for differences across the programs based on the information collected. This warrants further investigation.

### Directions for future research

Based on discussions at the Cochrane 2013 Colloquium meeting and by having taken a more in-depth look at the methods employed by the network members, various organizations employ different measures for their program. The impact of a RR used to inform health care decisions remains unknown as few programs have measured it. Impact measurement, although complicated, should be encouraged. It would be important to capture metrics related to, for example, how often the report was used to inform a decision, policy or debate; cost savings; harms reduction; and/or how often, and in what manner it was portrayed in the media. The results of our analysis highlight the reality that RR products provide flexibility and are time sensitive. Methodological research should be undertaken to develop taxonomy of types of RRs and understand the strengths and limitations of the various forms of RRs in keeping with the volume and type of evidence identified, as well as the level of synthesis performed. How the findings are reported may impact health care decisions and understanding the extent of bias [8]. Future research also can explore the methods used across a wide range of RRs produced by various organizations to assess how they align with the Preferred Reporting Items for Systematic Reviews and Meta-Analyses (PRISMA) guidelines and a measurement tool to assess systematic reviews (AMSTAR). This research will be undertaken by one of our coauthors, who is pursuing funding to assess a large sample of RRs from a variety of organizations, as part of her doctoral studies. The findings will provide some insight on the similarities and differences between a RR and a traditional SR to allow for a better understanding on the production of a RR in a shorter turnaround time and with fewer resources, as well to the extent a SR may be of merit. In addition, future studies should investigate potential trends of RR programs according to numerous characteristics, such as geography, organization type, and target audience. Moreover, a qualitative research study of evidence-based health researchers, including RR producers and health care decision makers, should investigate the feasibility and desire for an extension to the PRISMA guidelines to incorporate RR methodologies [9]. At a minimum, reporting guidelines can increase the transparency of RRs.

## Conclusions

The processes and methods for 29 international RR programs were reviewed and compared for greater insight on the different forms of RRs. Although numerous RRs focus on questions of clinical effectiveness, efficacy and cost-effectiveness, there is no single cohesive method to conduct a RR. In addition to the turnaround time, the resources available, the complexity and sensitivity of the research topics, and permission from the decision maker have an impact on the types of RR produced and their dissemination. Future studies should evaluate the appropriateness of the different RR methods and reporting for health care decisions, how the methods used across an array of RRs align with the PRISMA guidelines and AMSTAR tool, and the relevance of reporting guidelines for RRs to improve their transparency.

## Additional files

Additional file 1: Table S1. Data collection form for methods and processes of rapid review programs. Additional file 2: Table S2. Definitions and purpose of rapid reviews. Additional file 3: Table S3. Types of and dissemination tools for rapid reviews. Additional file 4: Table S4. Rapid review methods.

## References

[1] Ganann R, Ciliska D, Thomas H (2010). Expediting systematic reviews: methods and implications of rapid reviews. Implement Sci..

[2] Watt A, Cameron A, Sturm L, Lathlean T, Babidge W, Blamey S (2008). Rapid reviews versus full systematic reviews: an inventory of current methods and practice in health technology assessment. Int J Technol Assess Health Care.

[3] Khangura S, Polisena J, Clifford TJ, Farrah K, Kamel C (2014). Rapid review: an emerging approach to evidence synthesis in health technology assessment. Int J Technol Assess Health Care.

[4] Cameron A, Watt A, Lathlean T, Sturm T. Rapid versus full systematic reviews: an inventory of current methods and practice in health technology assessment. ASERNIP-S report no. 60 [Internet]. Adelaide (South Australia): Australian Safety & Efficacy Register of New Interventional Procedures – Surgical (ASERNIP-S); 2007 Jul. [cited 2012 Mar 1]. Available from: http://www.surgeons.org/media/297941/rapidvsfull2007_systematicreview.pdf

[5] Khangura S, Konnyu K, Cushman R, Grimshaw J, Moher D (2012). Evidence summaries: the evolution of a rapid review approach. Syst Rev.

[6] Harker J, Kleijnen J (2012). A methodological exploration of rapid reviews in Health Technology Assessments. Int J Evid Based Healthc.

[7] Hailey D, Corabian P, Harstall C, Schneider W (2000). The use and impact of rapid health technology assessments. Int J Technol Assess Health Care.

[8] Gough D, Thomas J, Oliver S (2012). Clarifying differences between review designs and methods. Syst Rev.

[9] Shamseer L, Moher D, Clarke M, Ghersi D, Liberati A (deceased), Petticrew M, et al. Preferred reporting items for systematic review and meta-analysis protocols (PRISMA-P) 2015: elaboration and explanation. BMJ 2015;349:g7647 10.1136/bmj.g7647 25555855

