# Supplementary material for: Rapid review programs to support health care and policy decision making: a descriptive analysis of processes and methods
Source: Syst Rev. 2015 Mar 14;4:26. doi: 10.1186/s13643-015-0022-6 (PMC4407715; doi:10.1186/s13643-015-0022-6)
Supplement: Additional file 1: Table S1. — Data collection form for methods and processes of rapid review programs. [file 13643_2015_22_MOESM1_ESM.docx]

**Additional file 1: Table S1: Data Collection Form for Methods and Processes of Rapid Review Programs**

| **Rapid Review Methods and Process** | **Rapid Review Steps** | **Rapid**  **Review**  **Program** | |
| --- | --- | --- | --- |
| Rapid Review definition |  |  | |
| Topic selection | Request submitted to rapid review producer |  | |
|  | Alternative topic selection (i.e., process that selects a rapid review topic other than a customer request) |  | |
|  | Purpose of rapid review report |  | |
| Education session with client/funder |  |  | |
| Protocol development | Title, project scope and research questions established |  | |
|  | Type of research question(s) |  | |
|  | Registration of protocol on PROSPERO/internally/publicly |  | |
| Report production | Literature search strategy |  | |
|  | Additional databases searched (e.g., EMBASE, CINAHL, PsycINFO) and automatic search updates |  | |
|  | Grey literature§ searched |  | |
|  | Search primary studies |  | |
|  | Search timeframe |  | |
|  | Language restrictions |  | |
|  | Search strategy peer review mandatory |  | |
|  | Study selection (one researcher) |  | |
|  | 2^nd^ independent researcher |  | |
|  | Data abstraction (one researcher) |  | |
|  | 2^nd^ independent researcher |  | |
|  | Level of extraction |  | |
|  | Number of included outcomes |  | |
|  | Evidence synthesis |  | |
|  | Narrative summary |  | |
|  | Meta-analysis |  | |
|  | Economic evaluation and∕or modelling |  | |
|  | Critical appraisal of studies (one researcher) |  | |
|  | 2^nd^ independent researcher |  | |
|  | Tool used for critical appraisal |  | |
|  | Industry input sought (i.e., solicitation of unpublished or cost data and feedback on draft report from industry) |  | |
|  | Context and policy issues/implications addressed |  | |
|  | Internal review of draft report |  | |
|  | External review of draft report |  | |
|  | Development of ‘key messages’ to support dissemination |  | |
|  | Report template |  | |
|  | Inclusion of tables |  | |
|  | Inclusion of graphs |  | |
|  | Inclusion of legal disclaimer |  | |
|  | Report contains reference to rapid review methods used |  | |
|  | Report length |  | |
|  | Author guidelines for report preparation |  | |
| Report submission and dissemination | Report submission to requestor |  | |
|  | Feedback of rapid review report and process sought from requestor |  | |
|  | Turnaround time from protocol preparation to report submission to requestor |  | |
|  | Report disseminated beyond original requestor |  | |
|  | Knowledge translation support  and/or dissemination tools |  | |
| Report publicly available | Report posted on web site |  | |
|  | Empty reviews* |  | |
| Report types produced by rapid review program |  |  | |
| Website** |  |  | |
| *Empty reviews: Reports where no evidence that met the selection criteria and are publicly available on the website. | | |  |
| ∞Literature timeframe can be adjusted as needed. | | |  |
| ^§^ Grey literature: includes reports and government information that are not published commercially and that are inaccessible via bibliographic databases. | | |  |
| ** Website where the methodology is posted | | |  |
