# Supplementary material for: Rapid review programs to support health care and policy decision making: a descriptive analysis of processes and methods
Source: Syst Rev. 2015 Mar 14;4:26. doi: 10.1186/s13643-015-0022-6 (PMC4407715; doi:10.1186/s13643-015-0022-6)
Supplement: Additional file 2: Table S2. — Definitions and purpose of rapid reviews. [file 13643_2015_22_MOESM2_ESM.docx]

**Additional file 2: Table S2: Definitions and Purpose of Rapid Reviews**

| **Rapid Review Element** | **Description** |
| --- | --- |
| Definition (n=25) | Accelerated:   - accelerated evidence synthesis/ abbreviated systematic review methods - expedited evidence review/ report or jurisdiction   Condensed:   - broad brush review of review-level literature - condensed/ restricted time frame - not an in-depth analysis of the data - not a comprehensive systematic review - short reports based on limited search of electronic databases - succinct review - systematic review methodology within a restricted timeframe   Focused:   - detailed and objective assessment and synthesis of the current research evidence - focused on specific technology - limited in scope/ methodology - pre-reimbursement single technology assessment for hospital - single technology assessment   Form of evidence synthesis:   - review conducted by one reviewer with no meta-analysis, modeling or GRADE - review of full HTA reports from other organizations - review of medical method (non-drug) the use of which hospitals are planning to introduce or spread - summary of available data/ summary of published literature/ summary of evidence - listing of potentially relevant information - overview of existing evidence/ current state of the evidence - provides a detailed and objective assessment and synthesis - provides ‘best evidence’ - provides evidence-based answers - rapid response provides access to up-to-date research evidence - support evidence-informed programs, service delivery and advocacy - synthesis of data - systematically review and summarize existing evidence   Modified:   - does not formally appraise the methodological quality of the included studies - knowledge synthesis in which components of the systematic review process are simplified or omitted to produce information in a timely manner - modified systematic review - no formal appraisal of methodological quality - systematic review methodology simplified or omitted - trade-off between robust methodology and the need for rapidity   Tailored:   - tailored to decision makers/ provide recommendations to decision makers |
| Purpose (n=26) | Assessment of impact and effectiveness:   - assess impact and effectiveness of intersectoral action on social determinants of health and health equity   Complement and clarification:   - complement discussions with other health service staff and manufacturers - improve understanding on a particular issue   Inform decision making and/or implementation :   - inform a policy decision on drugs - inform decision making regards treatment/equipment/services funding and policy and program development - inform decision making on coverage, formulary, policy, purchase, reimbursement of treatments, investment/disinvestment decision making, clinical practice, and/or regulatory status and coverage - inform decision making for health care organization - inform issue briefings with time pressures - inform stakeholders discussions and as input to internal projects - inform/support decision-makers and professionals faced with time constraints - provide evidence-based data to help clients make decisions on wide range of health and social science topics, including suicide prevention, conceptions of masculinity, obesity, and musculoskeletal injuries - support clinical decision making, implementation and clinical/operational effectiveness and efficiency - support evidence-informed programs, service delivery and advocacy   Provide evidence:   - provide access to research evidence for community-based organizations - provide general view on available published research evidence - provide support for evidence-based decision making - provide timely and accurate syntheses of targeted health care topics to clinicians, managers and policymakers, as they work to improve the health and health care of patients |
