# Supplementary material for: Rapid review programs to support health care and policy decision making: a descriptive analysis of processes and methods
Source: Syst Rev. 2015 Mar 14;4:26. doi: 10.1186/s13643-015-0022-6 (PMC4407715; doi:10.1186/s13643-015-0022-6)
Supplement: Additional file 3: Table S3. — Types of and dissemination tools for rapid reviews. [file 13643_2015_22_MOESM3_ESM.docx]

**Additional file 3: Table S3: Types of and Dissemination Tools for Rapid Reviews**

| **Rapid Review Element** | **Description** |
| --- | --- |
| Types of reports produced | - Abstract - Annotated bibliography - Clinical effectiveness review - Clinical practice guidelines - Evidence advisory - Evidence brief - Evidence inventory - Evidence notes - Evidence review - Expedited systematic review - Extensive review - Health technology assessment summary - Jurisdictional review - MUMM report - Peer-reviewed summary with critical appraisal - Rapid annotated bibliography - Rapid evidence maps - Rapid evidence report - Rapid health technology assessment - Rapid network meta-analysis - Rapid report - Rapid response - Rapid review - Rapid review of primary studies - Rapid review of systematic review - Rapid systematic reviews - Reference list - Research alert - Review of reviews - Scoping review - Single technology assessment - Snapshot review - Special report - Summary of abstracts - Summary with critical appraisal - Systematic review/meta-analysis - Time-sensitive review |
| Tools used to disseminate final report | - Academic or policy conference or forum - Conference presentations - Educational materials for selected medical technology topics - Email distribution - Guidance documents - Listservs - Organization intranet - Poster presentation - Posting on organization website - Posting on requestor website - Presentations - Publication in an online review summary database - Publication in peer-reviewed journal - PubMed Index - Recommendations memos - Reports are disseminated by requestors - Reports in brief - Social media: Twitter; RSS - Stakeholder meeting or workshop - Summaries/executive summaries/evidence summaries - University memo - Update in weekly or monthly e-newsletter - Video summary - Webinar - Workshop |

GRADE: HTA: health technology assessment
