# Supplementary material for: Rapid review programs to support health care and policy decision making: a descriptive analysis of processes and methods
Source: Syst Rev. 2015 Mar 14;4:26. doi: 10.1186/s13643-015-0022-6 (PMC4407715; doi:10.1186/s13643-015-0022-6)
Supplement: Additional file 4: Table S4. — Rapid review methods. [file 13643_2015_22_MOESM4_ESM.docx]

**Additional file 4: Table S4: Rapid Review Methods**

| **Rapid Review Methods** | **Description** |
| --- | --- |
| Data sources used | - AHRQ - BC/BS HTA program - BMJ Clinical Evidence - CADTH - Campbell Library - CDSR - CINAHL - Cochrane Library - Cochrane Systematic Reviews - CRD database - Dalhousie University’s Prowler database - DARE - EBM Reviews - ECRI, Embase - FDA - GIN - Google Scholar - Guidelines.gov - Hayes Inc. - Healthevidence.org - Healthsystemsevidence.org - HTA - Joanna Briggs - MEDLINE - NHS EED - NICE - Professional society guidelines databases - Project Cork - Psychology and Behavioral Sciences Collection - PsycINFO - PubMed - Scopus - Soc Sci Index - Social sciences abstracts - Social Services/Social Work Abstracts - Sociological abstracts - TA and ESP programs - Taiwan Periodical Literature System, Trial registries - TRIP database - Tufts CER - USPSTF - Veterans Administration - Washington State HTA program - Web of Science |
| Critical appraisal tools used | - AGREE or AGREEII - AHRQ or Cochrane manual - AMSTAR - Cochrane Risk of Bias tool - Critical Appraisal Skills Programme - Critical Review Form-Qualitative Studies 2.0 - Downs and Black - Drummond tool - Effective Public Health Practice Project - GRADE - INAHTA Qualitative Study Tool - Jadad - McMaster Critical Review Form - Newcastle Ottawa Scale - Oxman & Guyatt scale - QUADAS - Qualitative Assessment and Review Instrument - Quality assessment tool for quantitative studies for public health nursing interventions - Standard IQWiG methods - Tool by Mays and Pope - Tool dependent on study design - Tools by Guyatt et al. - Tools on Healthevidence.ca - Tools used in Health Evidence Wales - UK Effective Public Health Practice Project Quality Assessment Tool - USPSTF/CRD methods |
